# Doxorubicin-Induced Vascular Toxicity – Targeting Potential Pathways May Reduce Procoagulant Activity

**DOI:** 10.1371/journal.pone.0075157

**Published:** 2013-09-20

**Authors:** Irit Ben Aharon, Hadas Bar Joseph, Moran Tzabari, Boris Shenkman, Nahid Farzam, Mattan Levi, Ruth Shalgi, Salomon M. Stemmer, Naphtali Savion

**Affiliations:** 1 Institute of Oncology, Davidoff Center and Rabin Medical Center, Petah-Tiqva and Sackler Faculty of Medicine, Tel Aviv University, Tel Aviv, Israel; 2 Department of Cell and Developmental Biology, Sackler Faculty of Medicine, Tel Aviv University, Tel Aviv, Israel; 3 Amalia Biron Research Institute of Thrombosis and Hemostasis, Sheba Medical Center, Tel Hashomer, Israel; 4 Goldschleger Eye Research Institute and Department of Human Molecular Genetics and Biochemistry, Sackler Faculty of Medicine, Tel Aviv University, Tel Aviv, Israel; Virginia Commonwealth University, United States of America

## Abstract

**Introduction:**

Previous study in mice using real-time intravital imaging revealed an acute deleterious effect of doxorubicin (DXR) on the gonadal vasculature, as a prototype of an end-organ, manifested by a reduction in blood flow and disintegration of the vessel wall. We hypothesized that this pattern may represent the formation of microthrombi. We aimed to further characterize the effect of DXR on platelets’ activity and interaction with endothelial cells (EC) and to examine potential protectants to reduce DXR acute effect on the blood flow.

**Methods:**

The effect of DXR on platelet adhesion and aggregation were studied *in* *vitro*. For *in* *vivo* studies, mice were injected with either low molecular weight heparin (LMWH; Enoxaparin) or with eptifibatide (Integrilin^©^) prior to DXR treatment. Testicular arterial blood flow was examined in real-time by pulse wave Doppler ultrasound.

**Results:**

Platelet treatment with DXR did not affect platelet adhesion to a thrombogenic surface but significantly decreased ADP-induced platelet aggregation by up to 40% (p<0.001). However, there was a significant increase in GPIIbIIIa-mediated platelet adhesion to DXR-exposed endothelial cells (EC; 5.7-fold; p<0.001) reflecting the toxic effect of DXR on EC. The testicular arterial blood flow was preserved in mice pre-treated with LMWH or eptifibatide prior to DXR (P<0.01).

**Conclusions:**

DXR-induced acute vascular toxicity may involve increased platelet–EC adhesion leading to EC-bound microthrombi formation resulting in compromised blood flow. Anti-platelet/anti-coagulant agents are effective in reducing the detrimental effect of DXR on the vasculature and thus may serve as potential protectants to lessen this critical toxicity.

## Introduction

Doxorubicin (DXR), an anthracycline that is a cornerstone of many chemotherapeutic protocols, is used for treating a wide spectrum of malignancies. Several studies have established the deleterious effect DXR exerts on the vasculature *in vivo* [1-3]. and *in vitro* [4-8], although the mechanism that lies at the core of DXR-induced vascular toxicity remains obscure.

In a former study, we have established in a mouse model, a platform of innovative high resolution *in vivo* molecular imaging, suitable for capturing of vessels’ characteristics, arterial blood flow and organs blood volume that enables prolonged acute real-time detection of chemotherapy-induced effects in the same individuals. Following DXR administration, we observed an acute reduction in gonadal and femoral blood flow. Intravital imaging of gonadal and femoral microvasculature was obtained by fluorescence optical imaging system, equipped with a confocal fiber microscope (Cell-viZio) demonstrated that 3 minutes after DXR injection the wall of the blood vessels became irregular and the fluorescence signal displayed in the small vessels was gradually diminished, an indication of an immediate vessels’ injury [3]. Based upon previous studies demonstrating DXR-induced apoptosis in EC resulting in hypercoagulable state [9,10], we hypothesized that the pattern of the immediate vessel disintegration following DXR treatment may represent the formation of microthrombi on a thrombogenic vessel wall. That led us to further investigate the role of platelets in this pathogenesis.

It has been reported that DXR affected the procoagulant activity of macrophages and EC *in vitro*, altered the membrane fluidity and facilitated the activation of tissue factor (TF) [11,12]. In cell culture exposed to DXR, thrombin generation was significantly elevated and mRNA of TF was upregulated compared with other classes of chemotherapy, implying of the procoagulant traits of DXR [9-13]. Most of the DXR-induced thrombin generation can be attributed to increases in phosphatidylserine (PS) exposure; however, TF activation also appears to play a role [9]. DXR impaired the endothelium-based protein C anticoagulant pathway in a culture of vascular EC [4]. The effect of DXR on platelets had been studied *in vitro* where it has been shown to directly induce platelet cytotoxicity through reactive oxygen species (ROS) generation, decreased glutathione levels, and protein thiol depletion that may contribute to the development of thrombocytopenia following treatment [10,14].

Based upon our previous intravital imaging, we aimed to further characterize the immediate effect of DXR on the morphology of the regional vasculature as well as platelets’ function, and to examine the use of anti-thrombotic and anti-platelet agents to reduce the effect of DXR on the acute reduction in blood flow. It has been formerly demonstrated that glycoprotein (GP) IIb/IIIa antagonists, besides there inhibition of platelet aggregation, at high local concentrations may enhance disaggregation of platelet aggregates by disrupting the fibrinogen-bridged platelet-platelet binding [15,16]. Low molecular weight heparin (LMWH) was shown to reduce the vascular toxicity and ischemia in liver and kidney vessels in DXR-exposed rats [17,18]. We therefore evaluated the therapeutic efficacy of these clinically used anti-platelet and anti-thrombotic agents, the GP IIb/IIIa inhibitor eptifibatide, (Integrilin^©^), and the LMWH enoxaparin (Clexane^©^), respectively, on the acute effect of DXR on vascular blood flow, since these widely used agents, which have been proven to be clinically safe may confer a significant potential in lessening the critical vascular toxicity induced by DXR.

## Methods

### Animals

ICR mature male mice (7-8 weeks old; Harlan Laboratories, Jerusalem, Israel) were housed in air conditioned, light controlled animal facilities of the Sackler Faculty of Medicine, Tel-Aviv University. Following experimental procedures mice were sacrificed using CO_2_ chamber. Animal care and all experiments were in accordance with the institutional guidelines and were approved by the Institutional Animal Care and Use Committee of the Sackler Faculty of Medicine, Tel-Aviv University; ID number M-09-049.

### Chemotherapy

DXR (8mg/kg, Adriamycin; Teva, Israel) or saline, were injected intravenously (IV) into the tail vein at a volume of 100 µl.

### Ultrasound imaging

The molecular bioimaging platform enables real-time evaluation of the same individual over time, and hence each mouse serves as its own control.

#### Imaging of animals

Mice were anesthetized with isoflurane (Nicholas Piramal India Limited, India; 5% in oxygen for induction, 1–3% for maintenance at a rate of 1 liter/minute). Hemodynamic measurements were performed continuously throughout the experiments. Mice were positioned on a MousePad (part of the VisualSonics Vevo Integrated Rail System II) equipped with integrated heater and ECG electrodes (IndusInstruments, Houston, TX). Four legs were secured to ECG pads, with mediation of electrode cream (Signa cream; Parker Laboratories Inc., Fairfield, NJ, USA), to allow continuous monitoring of respiration rate. Respiration rate was maintained constant (varied between 20-40 breaths/minute in the cohort), body temperature was maintained at 37.5°C.

A 30-gauge, 1/2-inch needle attached to 1 ml syringe was inserted into the tail vein for IV administration of both contrast agent and either DXR or saline. Dorsal and groin fur was removed by a depilatory cream (Veet, Reckitt Benckiser, Bristol, UK). Pre-warmed ultrasound gel (Aquasonic, Parker Laboratories Inc, Fairfield, NJ, USA) was used as a coupling agent between the ultrasound scan-head and the skin.

Mice gonads or femoral arteries were viewed by the color mode of the high-resolution ultrasound (Vevo 2100; Visual Sonics, Toronto, Canada), with the transducer (MicroScan MS 550D; 22-55 MHz) held immobilized, in-position by the VisualSonics Vevo Integrated Rail System II.

To reduce variability, image parameters remained constant throughout the experiment (i.e., focus and depth optimized for each animal at the beginning of the experiment and the point of monitoring was fixed through the entire experiment). The same scan plane approximation, determined by anatomic markers, was used in all experiments.

#### PW Doppler measurement of blood flow in testicular vessels

Testicular blood volume and flow was viewed at the PW Doppler mode using the appropriate VisualSonics software. Following a short stabilization period, a baseline femoral or testicular arterial blood flow was recorded during a 50 second cine loop and quantified by analyzing the Velocity-Time Integral (VTI) [3,18-21]. When the PW Doppler mode curve is integrated, it yields a VTI that indicates the distance the blood travels during a certain cardiac circle. Mice were injected with either LMWH (Enoxaparin; Clexane©, Sanofi Aventis, Israel; 100 µg/mouse, SC; n=8) 24 hrs and 1 hour prior to DXR treatment, or with eptifibatide (Integrilin©, Millennium Pharmaceuticals, Inc., Cambridge, Massachusetts, USA ; 75 µg/mouse; IV; n=9) 90 minutes prior to DXR treatment. Testicular arterial blood flow was examined and analyzed prior and immediately following DXR treatment. The reference treatments were injections of DXR alone (n=8), LMWH and saline (n=6) or eptifibatide and saline (n=5), respectively. The arterial blood flows were monitored continuously for 20 minutes and analyzed at various time points post DXR injection.

#### Image analysis

Acquired contrast (ovarian blood volume) and PW Doppler (testicular and femoral arterial blood flow) cine loops were digitally stored and pooled for off-line analysis.

### Platelet aggregation

Blood samples were obtained from healthy volunteers that provided their written informed consent to participate in this study as approved by the Sheba Medical Center Institutional Review Board.

Blood was collected routinely into 3.2% sodium citrate (9:1, blood: anticoagulant ratio). Platelet rich plasma (PRP) was prepared by centrifugation at 140 g for 10 minutes at room temperature. Light transmission platelet aggregometry was induced by ADP (5 µM) and the aggregation was recorded using the aggregometer device (Helena Laboratory; PACKS-4, Beaumont, TX, USA) for 6 minutes.

### Platelet adhesion

The Impact-R [Cone and plate(let) analyzer (CPA)] (Matis Medical, Brussels, Belgium) is designed to evaluate platelet adhesion under flow conditions [22]. Samples of sodium citrate anti-coagulated whole blood (0.13 ml) were placed on polystyrene wells and subjected to flow at 1800 s^-1^ for 2 minutes. Immediately upon blood contact with the polystyrene surface plasma proteins such as von Wileibrand Factor and fibrinogen adhere to the surface and makes it a thrombogenic surface upon which platelets can adhere and aggregate. At the end of the 2 minutes flow wells were then thoroughly washed with water, stained with May-Gruenwald stain and analyzed by the Impact-R image analyzer system connected to a microscope. Platelet deposition was determined by measuring the percentage of the well surface covered with platelets (surface coverage) and the average size of the adherent particles in µm^2^.

### Endothelial cell culture

Adult bovine aortic EC were prepared from the bovine aortic arch obtained from the slaughter house as approved by the Sheba Medical Center Institutional Review Board, and grown as previously described [23]. Briefly, endothelial cell cultures were grown at 37°C in 10% CO_2_ in Dulbecco’s modified Eagle’s medium (DMEM) supplemented with 5% calf serum, 5% (v/v) heat-inactivated fetal calf serum, 2 mM L-glutamine, 100 U/ml penicillin, 100 g/ml streptomycin, and 12.5 U/ml nystatin. All media products were purchased from Biological Industries Ltd (Beit Haemek, Israel). Human recombinant FGF-2 (a generous gift from Amgen, Boulder, CO) was diluted in DMEM supplemented with tissue culture grade bovine serum albumin (Sigma, St. Louis, MO) and added to the cultures every other day at a concentration of 3ng/ml until cultures were nearly confluent. For experiments cultures were grown in tissue culture 4-well plates (Nunc, Roskilde, Denmark) and upon reaching confluence used for experiments.

### Platelet adhesion to endothelial cells

Confluent EC in growth medium were pre-incubated with DXR (100 µM) for 4 hr then washed 3 times and whole blood (0.2 ml) was added. The blood was pre-incubated (15 min) without or with eptifibatide or with mouse monoclonal anti human CD154 (CD40L; Serotec, Raleigh, NC, USA) or mouse monoclonal anti human CD42b (GPIb; Santa Cruz Biotechnology, Dallas, TX, U.S.A.) or normal mouse IgG (Dako, Glostrup, Denmark) as control. The blood placed on the cell layer was subjected to flow for 5 minutes at 37°C under defined shear rates (750 s^-1^) created by a rotating Teflon cone specifically designed for the Cone and Plate (let) Analyzer (CPA) [22]. At the end of the incubation the cell layers were thoroughly washed with phosphate-buffered saline (PBS), fixed by exposure to 4% paraformaldehyde solution in PBS, adjusted to pH 7.4, kept at 37°C for 5 min, and then washed with PBS. The cell layer was further treated for 5 minutes with hydrogen peroxide 30%, washed 3 times with Tris-buffered saline (50 mM Tris, 150 mM sodium chloride, pH 7.6), and subjected to immunohistochemistry using a Histostain-Plus kit (Invitrogen, Fredrick, MD, USA) with aminoethyl carbazole as the substrate chromogen. The first antibody used to detect the adhered platelets on the EC monolayers was the specific monoclonal antibody against the platelet integrin CD41a (BD-Pharmingen, San Diego, CA, USA) diluted in 0.1% albumin solution/0.05% Tween-20 in PBS. Stained platelets were counted using an Olympus microscope (X10 magnification) connected to a camera and the CPA image-analysis system of the Impact-R (Matis Medical, Brussels, Belgium). The system picked 4 random fields with an area of 0.5 mm^2^ each for each well.

### Immunohistochemistry and confocal microscopy

Mice were scarified either one week or one month after administration of saline or DXR, tetses were excised and fixed in 4% paraformaldehyde, embedded in paraffin blocks and sectioned. For immunohistochemistry, sections were deparaffinized, rinsed in PBS and incubated for 1 hr with PBSTg (0.2% Tween-20, 0.2% gelatin in PBS), re-rinsed in PBS, blocked for 10 minutes in blocking solution (927B; Cell Marque, CA, USA) and incubated over-night at 4°C with rat polyclonal anti CD34 (1:200; Cedarlane, Ontario, Canada), which is a marker for EC. Sections were then rinsed in PBSTg (x3) and secondary antibody was applied (Alexa555-conjugated goat anti rat IgGs ; 1:400; Invitrogen, CA, USA). Hoechst 33342 was used for nuclei staining. Sections were rinsed in PBS (x3) and mounted with moviol (Sigma). Labeled sections were visualized and photographed by a confocal laser-scanning microscope (Leica TCS SP5; Mannheim, Germany), equipped with an argon-ion laser (458 nm, 476 nm, 488 nm, 496 nm, 514 nm lines), a diode-pumped solid state laser (516 nm line) and an UV diode laser. Water-immersion lenses (20× NA/0.7 and 63× NA/1.2) were used for all imaging.

### Statistical analysis

Each experiment was repeated at least three times. Differences in the intensity of labeling by CD34 of the various treatment groups were assessed using the two-way analysis of variance (ANOVA) test with a significance of P < 0.01. ANOVA with repeated measures was employed for assessing the degree of change in blood flow in the testicular blood flow experiment. Differences in platelet adhesion to EC were assessed by Kruskal-Wallis test (non-parametric ANOVA) Dunn’s multiple comparison test. Results were considered statistically significant at P<0.05.

## Results

### Effect of DXR on platelet aggregation and adhesion

**Figure 1:**
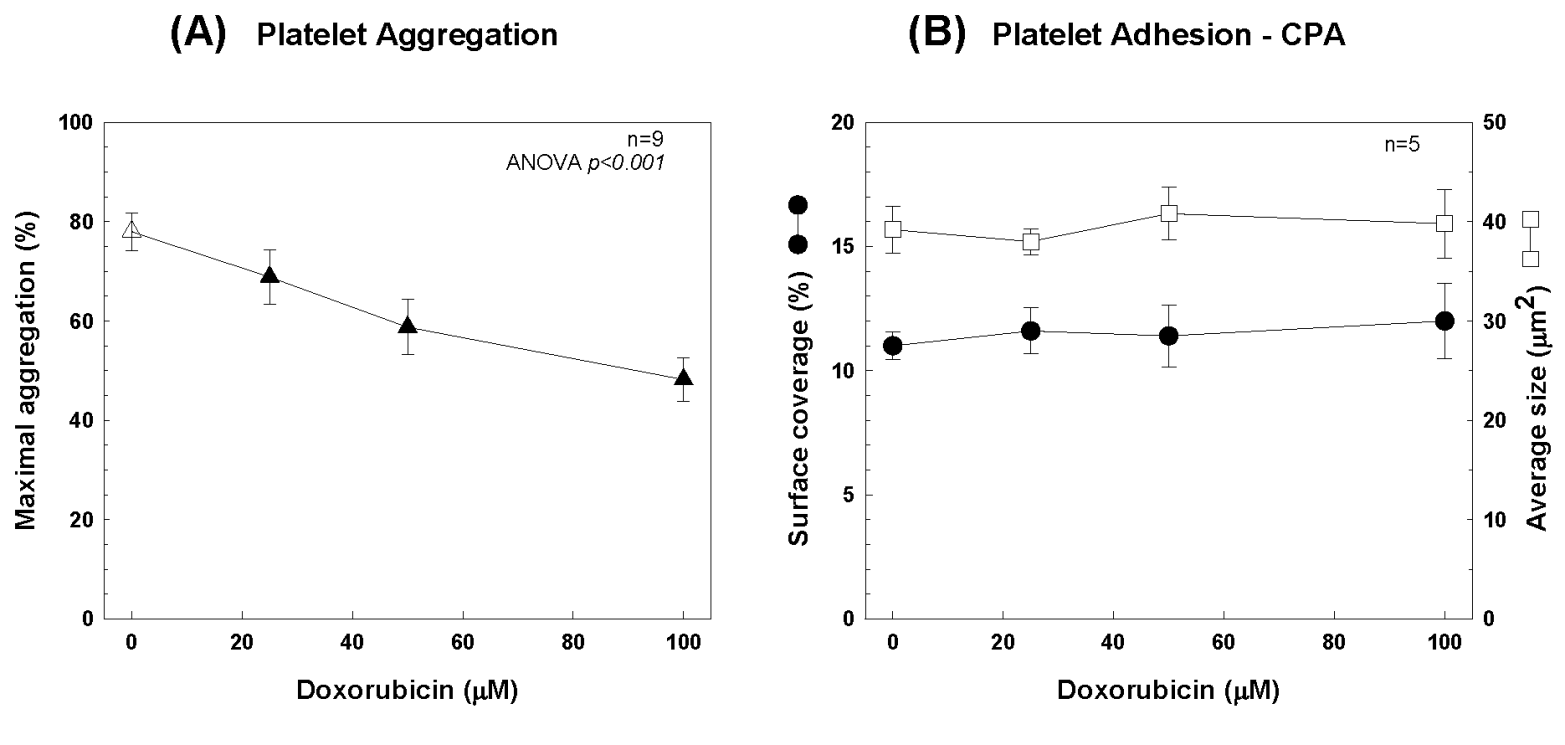
Effect of Doxorubicin on platelet aggregation and adhesion. (A) PRP was pre-incubated for 15 minutes with increasing concentrations of DXR then aggregation was induced by ADP (5 µM) and maximal aggregation is presented as mean ± SD; statistical analysis was tested by ANOVA (p<0.001; n=9). (B) Whole blood was pre-incubated for 15 minutes with increasing concentrations of DXR then subjected to the Impact-R test and both surface coverage and average size of the aggregates are presented as mean ± SD (n=5).

PRP was pre-incubated for 15 minutes with increasing concentrations of DXR, then platelet aggregation was induced by ADP (5 µM) (Figure 1A). A significant DXR dose-dependent decrease in platelet aggregation was observed reaching up to 40% inhibition at 100 µM (p<0.001).

### Platelet adhesion

Whole blood was pre-incubated for 15 minutes with increasing concentrations of DXR then subjected to the Impact-R test (Figure 1B). Both surface coverage and average size demonstrate no effect of DXR on platelet adhesion (p<0.001).

These experiments demonstrated the inhibitory effect of DXR on platelet aggregation but without any effect on platelet adhesion to a thrombogenic surface.

### Platelet adhesion to DXR-treated aortic EC

**Figure 2:**
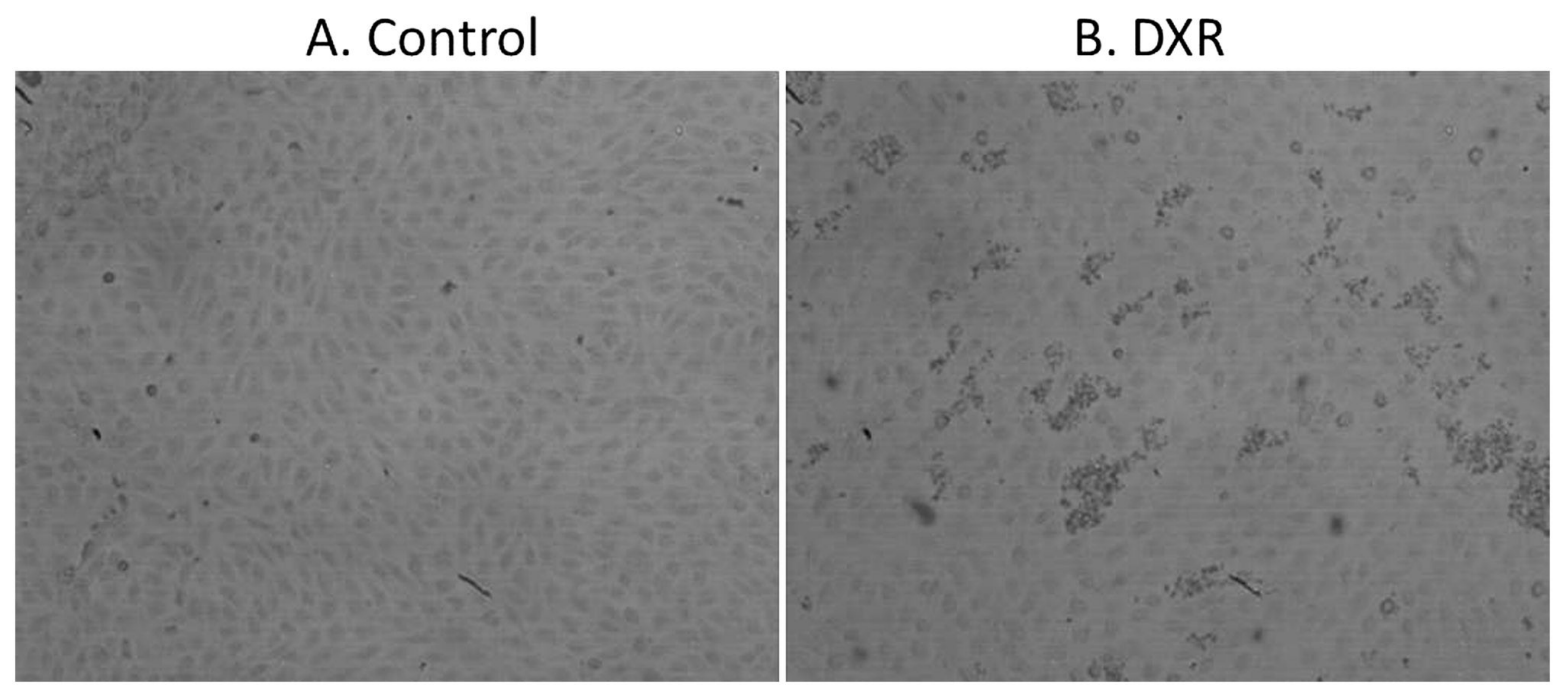
Platelet adhesion to DXR-treated aortic endothelial cells. Confluent endothelial cells were exposed to growth medium without (A) or with Doxorubicin (B; 100 µM) for 4 hr followed by exposure to whole blood for 5 minutes at 37°C under defined shear rates (750 s^-1^) and then fixed and platelets were stained by immunohistochemistry kit using monoclonal antibody against the platelet integrin CD41a.

Confluent EC were exposed to DXR (100 µM) for 4 hr followed by exposure to whole blood for 5 minutes at 37°C under defined shear rates (750 s^-1^) and then fixed and platelets were stained by immunohistochemistry kit using monoclonal antibody against the platelet integrin CD41a (Figure 2). There was a significant increase of 5.7-fold in platelet adhesion to EC treated with DXR compared to control none treated EC (40 ± 10 and 231 ± 40 platelet particles per 0.5 mm^2^, respectively; p<0.001; n=8) reflecting the toxic effect of DXR on EC (Table 1). Pre-incubation of the blood, prior to exposure to the EC, with the platelet aggregation inhibitor eptifibatide, blocking the integrin GPIIbIIIa, or with mAb against CD40L bound to platelet surface that may mediate platelet binding to EC, significantly reduced platelet adhesion to 55% and 81% of DXR treatment, respectively (p<0.001). Pre-incubation of the blood with either mAb against CD42b (platelet GPIb receptor) or non-specific mouse IgG (as control) did not cause any significant change in platelet adhesion to DXR-treated EC (Table 1).

**Table 1:** Platelet adhesion to DXR-treated EC.

**Treatment**	**Mean Objects Number**	**SD**	**n**	**% control**	**% DXR**	**DXR vs Control**	**Treatment vs DXR**
Control	40	10	8				
DXR	231	40	8	577		P<0.001	
DXR+eptifibatide	127	41	4		55		p<0.05
DXR+anti CD40L	188	42	4		81		p<0.05
DXR+Normal IgG	297	74	2		128		
DXR+anti CD42b	215	77	4		93		

EC were pretreated without (Control) or with DXR (100 µM) for 4 hr then washed 3 times and exposed to whole blood (0.2 ml) pretreated for 15 min with eptifibatide (20 µg/ml) or mAb against human CD154 (CD40L; 20 µg/ml) or mAb against human CD42b (GPIb; 20 µg/ml) or normal IgG (20 µg/ml) as control under flow conditions (750 s^-1^) for 5 min using the CPA technology. The EC surface was then washed and the adhered platelets were immune-labeled using the anti-platelet CD41a specific antibody and the Histostain-Plus staining kit. The number of objects (platelets) on the EC surface was quantitated by the Impact-R image analyzer. Significant differences were assessed by the Kruskal-Wallis test (1 way ANOVA – non parametric) Dunn’s Multiple comparison test. Results were considered statistically significant at P<0.05.

### The effect of anti-coagulant and anti-platelet agents on testicular arterial blood flow following DXR treatment

**Figure 3:**
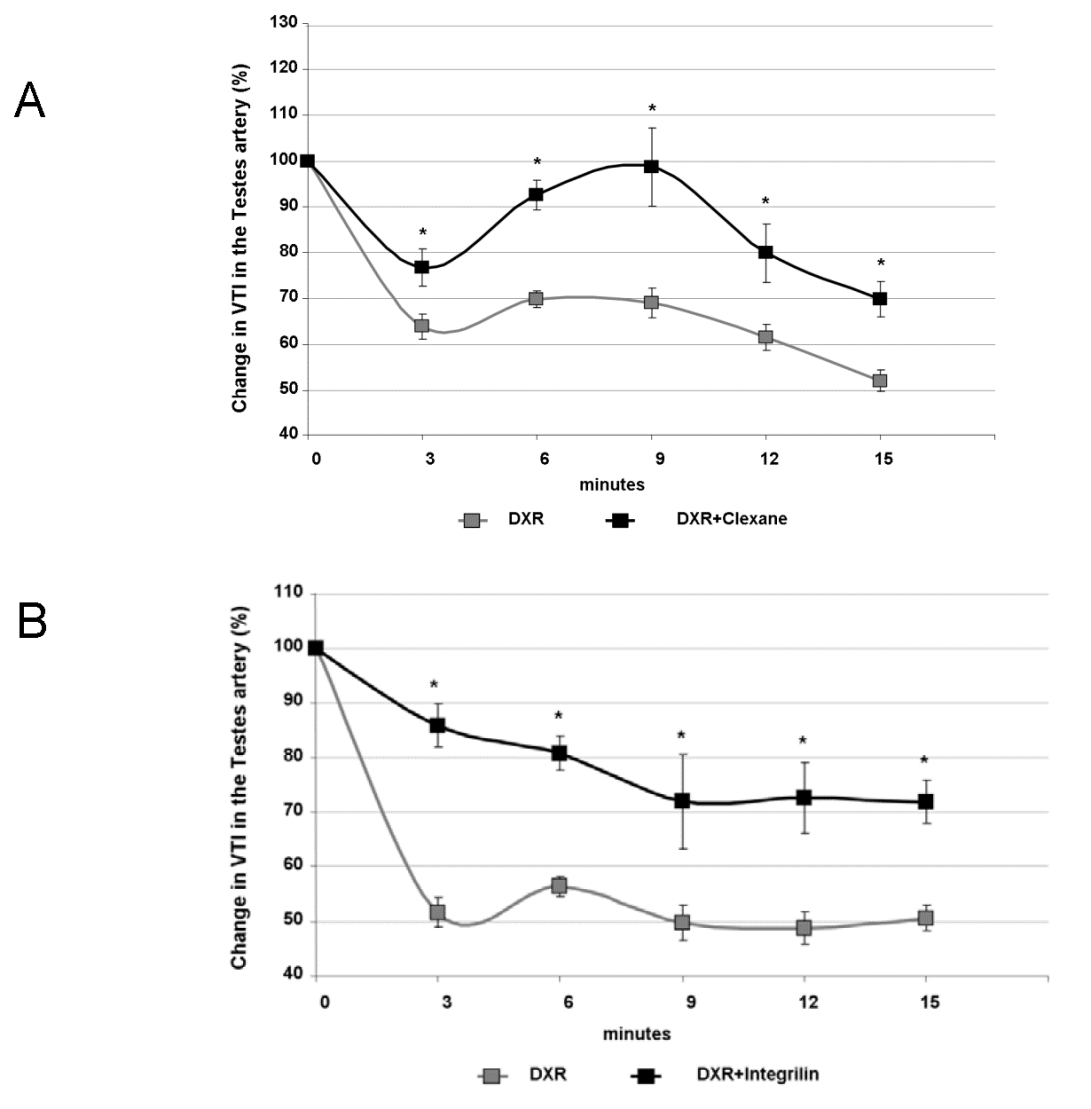
The effect of anti-coagulant and anti-platelet agents on testicular arterial blood flow following DXR treatment. Blood flow was measured by the PW Doppler mode and quantified by analyzing the VTI using the appropriate VisualSonics software. (**a**) Blood flow of testicular arteries was continuously monitored by PW Doppler in mice pre-treated with LMWH (clexane; 100µg/mouse), before and following DXR (8mg/kg BW; n=8, n=number of imaged arteries) injection and analyzed 3, 6, 9, 12 and 15 minutes afterwards. Graphic illustration of testicular arterial blood flow; points represent percent of control (dashed line; mean±SEM), (*)-significantly different from DXR treatment values (P<0.05). (**b**) Blood flow of testicular arteries was continuously monitored by PW Doppler in mice pre-treated with eptifibatide (integrilin; 75µg/mouse), before and following DXR (8mg/kg BW; n=9, n=number of imaged arteries) injection and analyzed 3, 6, 9, 12 and 15 minutes afterwards. Graphic illustration of testicular arterial blood flow; points represent percent of control (dashed line; mean±SEM), (*)-significantly different from DXR treatment values (P<0.05).

The potential protective effect of either LMWH or eptifibatide against the toxic effect of DXR on the testicular arterial blood flow in male mice was assessed by measuring VTI with the PW Doppler of the Vevo 2100 ultrasound device. A statistically significant difference (P<0.01) in testicular arterial blood flow between DXR treated mice and DXR and LMWH treated mice, was observed throughout all measured time points (Figure 3A), indicating that the testicular arterial blood flow was improved as a result of pre-treatment with LMWH. Administration of eptifibatide with DXR resulted in a potent attenuation of the DXR-induced reduction in testicular blood flow as well (Figure 3B).

### DXR mediates testicular vascular changes

**Figure 4:**
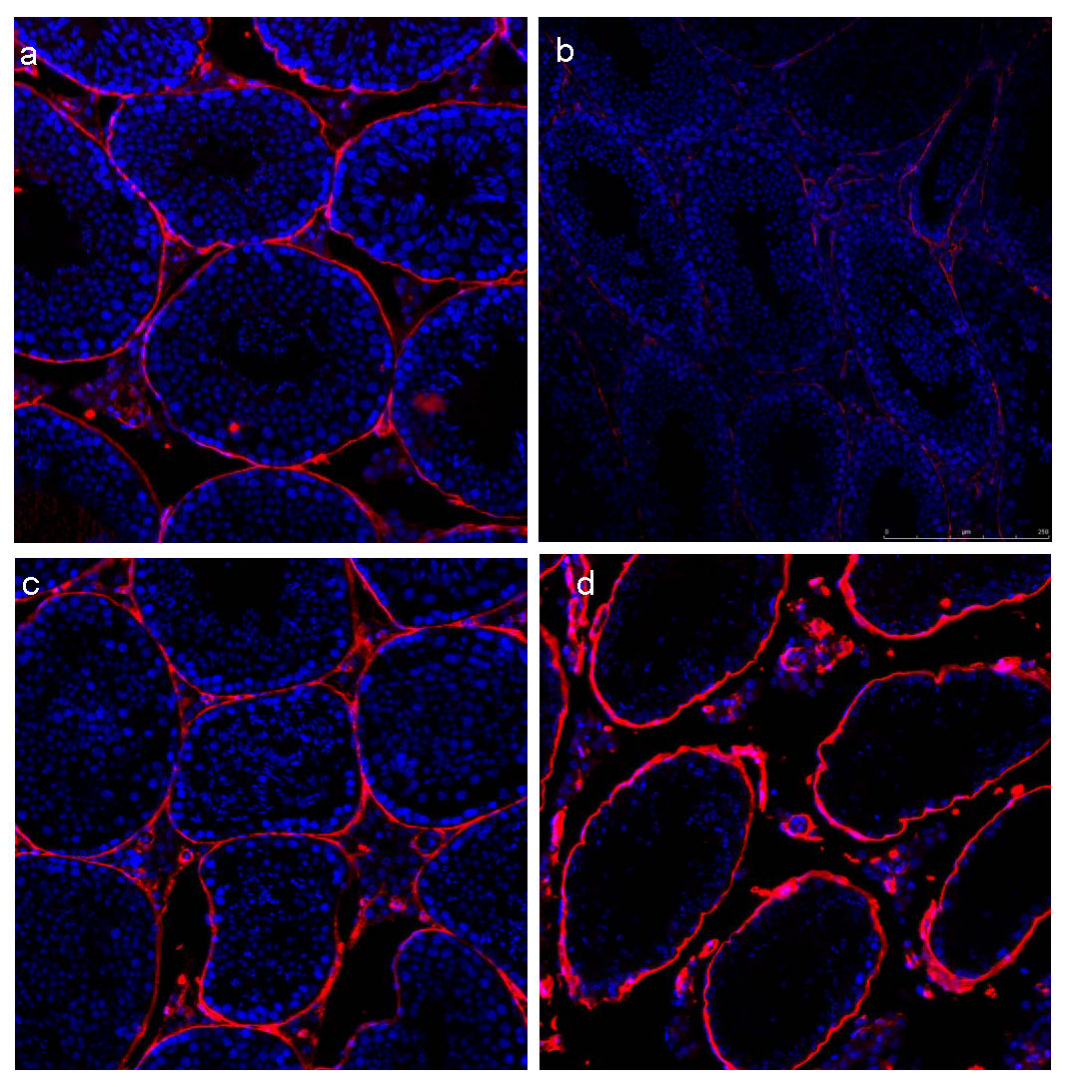
DXR induces testicular vascular changes. Histological sections of testes from saline or DXR (5 mg/kg) treated mice were immunohistochemically stained with Hoecst 33342 (nuclei labeling; blue) and anti CD34 primary antibody (1:200) followed by alexa555 anti-rat secondary antibody (red; 1:400). Saline (A,C) and DXR (B,D) at one week (A,B) or one month (C,D) after treatment. Bar=240µm.

In order to evaluate the sub-acute vascular changes in the testis following exposure to DXR, we employed CD34 as a marker for EC. Confocal scanning fluorescence microscopy revealed a dramatic decrease in testicular blood vessels one week after DXR injection (Figure 4b) as compared to control Saline injected (Figure 4a), while a significant increase in immunostaining was observed at one month (Figure 4d). This enhanced staining may be attributed to a recovery of the testicular stroma as CD34 has been shown to determine also testicular stromal cells (47), or to neovascularization as observed previously in ovaries subjected to chemotherapy (48) and particularly to DXR (49).

## Discussion

Our results indicate a potential link between DXR-induced acute vascular toxicity and activation of coagulation and platelet aggregation pathways. The two widely clinically used anti-coagulant and anti-platelet agents demonstrated a protective effect and minimized the acute reduction in vascular blood flow observed following DXR administration.

We had previously characterized the acute vascular effect of DXR using intravital imaging of gonadal and femoral microvasculature- a unique pattern of vessels’ injury few minutes after DXR injection. The wall of the vessels became irregular and the fluorescence signal displayed in the small vessels was gradually diminished [3]. This prompted us to study whether the vascular damage proceeds by activation of coagulation factors as well as platelet-endothelium interaction followed by aggregation that may mediate the toxic effect. Several studies have confirmed that DXR induces oxidative stress, a condition known to be toxic to EC, leading to loss of their barrier trait [14,24]. Few *ex vivo* studies have documented the vascular effect of DXR on tissues excised from DXR-injected animals. An impaired endothelial-dependent vasodilatory response to acetylcholine or adenosine was observed in rabbit and rat models with DXR–induced cardiomyopathy [25,26]. Brachial artery reactivity, a marker for endothelial vasodilatation function, detected by high-resolution ultrasound, was decreased in human patients that received at least 300 mg/m^2^ of DXR (or daunorubicin) compared to control patients [1]. Nevertheless, the cascade of events downstream to vessel injury has never been studied.

Several classes of chemotherapy have been shown to exert the activation of coagulation through the TF pathway [5,9,12,13]. It has been previously demonstrated that DXR alters the generation of plasma thrombin and consumption of prothrombin, and triggers the up-regulation of TF [13]. Recently Swystun et al. [27] conferred the activation of thrombin-antithrombin complex following anthracycline-based chemotherapy to the release of cell-free DNA (CFDNA) from chemotherapy-injured cells as an alternative mechanism for TF up-regulation [27,28]. In the present study our *in vitro* studies demonstrate the stimulatory effect of DXR on platelet adhesion to EC. This induced platelet-EC adhesion was mostly inhibited by eptifibatide and partially by anti-CD40L mAb, suggesting a major role for the platelet integrin GP IIb/IIIa and a minor role for CD40L in mediating the platelet-EC adhesion.

Former studies have demonstrated that DXR induces oxidative stress and apoptosis in platelets that may result in thrombocytopenia [10]. The pronounced acute vascular toxicity exerted by DXR may result in massive activation of coagulation factors due to enhanced endothelial injury; nevertheless, although there are former studies indicating that DXR induces hypercoagulable state, there is no clinical evidence for massive acute thrombotic events in the population treated with DXR. An explanation to this paradigm may be the paradoxical inhibitory effect of DXR on platelet aggregation on the one hand, while enhancing platelet adhesion to EC on the other hand. The outcome of these two reciprocal DXR-induced processes is probably the formation of endothelium surface-bound platelet aggregates (reflecting the increased platelet adhesion to DXR-exposed endothelium), which results in altered blood flow. However, the propagation of the aggregates and their stability appeared lower (reflecting the DXR inhibition of platelet aggregation but not adhesion). Indeed, the immediate effect of DXR as studied by intravital imaging demonstrated formation of unstable platelet micro-aggregates on the vessel wall that reduces blood flow but do not cause immediate complete blood vessel occlusion.

In a previous study we demonstrated that DXR treatment induces a hypercoagulable state that may manifest in the acute vascular dynamics. It is in agreement with other studies demonstrating that patients treated with chemotherapy have a 4.8-fold increased risk for venous thromboembolism (VTE) [29-31]. Furthermore, LMWH, which is widely used in cancer patients for secondary and primary prophylaxis, was shown to be superior in reducing the risk of VTE [32-34]. LMWH has been shown to confer a survival benefit in three studies that could not be linked to its anti-thrombotic activity but rather to anti-neoplastic traits [35-37] and is therefore the appropriate anti-coagulant as well as anti-metastatic agent. The anti-neoplastic effect may derive from potential crosstalk between heparin derivatives and the extracellular matrix by cell signaling molecules (as mucopolysaccharides) that affect tumor growth [38,39]. Furthermore, heparin was shown to inhibit heparanase activity, an essential activity for tumor neovascularization and growth [40]. LMWH have been shown to reduce oxidative stress metabolites derived from DXR-induced renal and cardiac toxicity [17,18]. In our study, we evaluated the potential role of LMWH in attenuating the vascular effect of DXR. LMWH diminished the phenomenon of reduced blood flow observed immediately following DXR administration. In a parallel setting we appraised its effect over time in female mice and observed a similar acute pattern of minimizing the vascular effect on the ovarian blood flow. Furthermore, LMWH also prevented the typical DXR-induced reduction in ovary size over time (at one month post treatment; data not shown). The mechanism by which LMWH attenuates the vascular effect of DXR *in vivo* was not explored in the present study. However, previous studies demonstrated the involvement of TF activation followed by thrombin generation in the formation and stabilization of a thrombus at the injured vessel wall [41]. We suggest that also in DXR-injured vessel wall the TF – thrombin axis is activated leading to further platelet activation resulting in stabilization of the growing aggregate and therefore, LMWH attenuates the vascular effect of DXR, probably by inhibition of thrombin activity.

The anti-platelet agent employed in our study was eptifibatide, a GP IIb/IIIa receptor antagonist. Eptifibatide had been incorporated into clinical practice following several studies which have indicated that higher levels of platelet GP IIb/IIIa receptor occupancy with eptifibatide are associated with improved myocardial perfusion and coronary blood flow among patients with ST-elevation myocardial infarction as well as improved cardiac outcomes among patients undergoing percutaneous cardiac intervention [42-44]. The integrin GP IIb/IIIa is the most abundant receptor on the platelet surface and is at its inactive form on the surface of quiescent circulating platelets [45,46]. Platelet activation leads to conformational change in the receptors, enabling GP IIb/IIIa binding to the bridging molecule, fibrinogen, thereby forming platelet aggregate [45-47]. High concentrations of GP IIb/IIIa antagonist may inhibit platelet aggregate formation as well as cause disaggregation of newly formed aggregates by disrupting platelet-fibrinogen binding [15,16]. Implementing eptifibatide in our *in vivo* platform yielded a potent protective effect on the abrupt reduction in testicular blood flow immediately after DXR administration and diminished completely the toxic effect, keener than LMWH effect.

Upon the literature and our former results, we focused on DXR-induced vascular toxicity in an end organ, and assessed the testicular architecture as well as vasculature. In order to evaluate the subacute phase as well as the recovery phase (i.e., long term effect) of vascular changes in the testes following exposure to DXR, we employed CD34, a marker for EC. Confocal scanning fluorescence microscopy revealed a dramatic decrease in testicular blood vessels one week after DXR injection, while recuperation of blood vessels manifested by enhanced immunostaining at one month. To note, the staining may be attributed also to a recovery of the testicular stroma [48], or to neovascularization as observed previously in ovaries subjected to chemotherapy [49] and particularly to DXR [50].

The acute reduction in gonadal blood volume and femoral arterial blood flow, and the impairment of the blood vessels’ wall which has been characterized in our former study has been DXR-unique and was not evident in other classes of chemotherapy. This may represent an acute universal DXR-related vascular toxicity, an initial event in organ injury, which may trigger the coagulation pathway and result in compromised blood flow due to microthrombi formation. In our former study we have established the pattern of DXR-induced acute vascular toxicity. In this current study we attempted to characterize potential key players in conveying this effect, and demonstrated that DXR affected both platelets as well as the vascular endothelium. Furthermore, we presented putative keys to reduce the detrimental effect of DXR on the vasculature by applying clinically used anti-platelet/anti-coagulant agents in our platform. Our study emphasized on the acute phase of DXR-induced vascular toxicity in view that this could be the seed for the formation of long-term cardiovascular diseases, and provided the initial evidence that these protectants may have a role in alleviating acute vascular toxicity. Implementing these agents which are clinically proven to be safe and efficient may be useful also in this “off-label” use and minimize the harmful effect of chemotherapy on the vasculature. Further studies are warranted to evaluate the short and long term effect of these protectants on oncological outcomes.
